# Effect of 2 weeks rest-pause on oxidative stress and inflammation in female basketball players

**DOI:** 10.1038/s41598-024-65309-5

**Published:** 2024-06-25

**Authors:** Justyna Cichoń-Woźniak, Joanna Ostapiuk-Karolczuk, Mirosława Cieślicka, Hanna Dziewiecka, Piotr Basta, Dariusz Maciejewski, Anna Skarpańska-Stejnborn

**Affiliations:** 1 Department of Biological Sciences, Faculty of Sport Sciences in Gorzów Wielkopolski, Poznań University of Physical Education, Estkowskiego 13, 66-400 Gorzów Wielkopolski, Poland; 2 Department of Human Physiology, Nicolaus Copernicus University Ludwik Rydygier Collegium Medicum in Bydgoszcz, Karłowicza 24, 85-092 Bydgoszcz, Poland; 3 Department of Physical Education and Sport, Faculty of Sport Sciences in Gorzów Wielkopolski, Poznań University of Physical Education, Estkowskiego 13, 66-400 Gorzów Wielkopolski, Poland

**Keywords:** Immunology, Physiology, Cytokines

## Abstract

Intense exercise leads to increased production of free radicals, resulting in an inflammatory response in athletes. For this reason, it was decided to investigate whether a single intensive exercise until exhaustion applied after a 2-week rest period would result in a violation of the pro-oxidant-antioxidant balance. Twenty-seven trained female basketball players (age: 16.55 ± 0.96 years, body mass: 66.40 ± 13.68 kg, height: 173.45 ± 5.14 cm) were enrolled to the study following the application of inclusion and exclusion criteria. Study was conducted at the end of the competitive training phase. Participants underwent incremental treadmill exercise, with blood samples collected before the test, immediately post-exercise, and after a 3-h restitution period. Total antioxidant capacity (TAC) levels increased significantly after exercise and remained unchanged after 3 h. Concentration of interleukin-10 (IL-10) and creatine kinase (CK) significantly increased after exercise and then decreased. Concentration of interleukin-2 (IL-2) was significantly reduced immediately and 3 h after exercise, while interleukin-13 (IL-13), interleukin-1α (IL-1α), and tryptophan (TRP) decreased 3 h after exercise. No significant changes were observed in other biochemical parameters. Obtained results show an increased antioxidant capacity which reduced oxidative stress and inflammation in response to intense exercise indicating that rested athletes have a high adaptation and elevated tolerance to effort.

## Introduction

Competitive athletes in the starting period are subjected to increased training loads to achieve the highest sports performance. Accumulation of loads is tolerated by their body thanks to the use of training periodization involving the application of alternating periods of intense exercise and rest. However, when the accumulation of training loads is not accompanied by a sufficient long rest period for regeneration, the overtraining syndrome can occur^1^.

Physical activity, especially of high intensity, induces damage to muscle fibers due to increased production of free radicals, which in turn leads to an inflammatory response^2,3^. Many factors have been shown to contribute to its development, including depletion of muscle glycogen, damage to contractile proteins by free radicals, and reduced mitochondrial capacity^4^.

Moderate level of production of reactive oxygen species (ROS) contributes to the physiological adaptation of skeletal muscles through the biogenesis of mitochondria, and the synthesis of antioxidant enzymes or stress proteins. Excessive production of free radicals leads to a shift in the redox balance towards oxidation and the formation of oxidative stress^5^, which significantly affects the dynamics of cellular and physiological processes, favoring the occurrence of several negative changes, including abnormalities in the functioning of the immune system, muscle proteolysis, or disturbances in iron metabolism. Oxidative stress also underlies the decline in performance and fatigue in athletes, which in turn leads to a weakening of the immune function and increased susceptibility to infections^6,7^.

One of the major consequences of pro-antioxidant imbalance is the shift of the pro-anti-inflammatory balance towards the pro-inflammatory one. In a typical inflammatory response model, interleukin-6 (IL-6) and tumor necrosis factor-alpha (TNF-α) are produced first, followed by an increase in interleukin-2 (IL-2) and interleukin-1α (IL-1α), which may lead to leukocytosis due to neutrophilia in the systemic circulation and immunosuppression^4^. Compensatory mechanisms appear in this very dynamic system, aimed at neutralizing the pro-inflammatory cytokine cascade through the release of anti-inflammatory cytokines, such as interleukin-10 (IL-10) and interleukin-13 (IL-13)^4,8^.

It is known that an optimal balance between training load and recovery is required to improve exercise performance^9,10^. Long-term imbalance can lead to fatigue, overload, and overtraining where, especially in overtraining, pro-antioxidant imbalance becomes a fixed pattern, leading to a dramatic decrease in the condition and deterioration of the athlete's health^11^. Elevation of pro-inflammatory cytokines following exercise may subsequently amplify oxidative stress, thereby exacerbating inflammation and perpetuating a vicious circle^12^. Therefore, monitoring changes in the time of regeneration may be predictive for planning appropriate training, which will significantly reduce the risk of injury and will not destabilize the sports form of athletes practicing competitive sports.

We hypothesized that a 2-week rest period might be sufficient to stabilize the pro-antioxidant balance, which may be associated with an exercise response not disturbed by inflammation. Accordingly, study aims at checking whether a 2-week rest period is sufficient to stabilize the pro-oxidant-antioxidant balance.

## Materials and methods

### Study design and participants

Twenty-seven female basketball players, who met the inclusion criteria of a minimum of 6 years of training experience, absence of iron homeostasis disturbances (e.g., iron deficiency, anemia), and regular menstruation cycles, took part in the study. Exclusion criteria included the presence of acute or chronic inflammation, pain disability, fever, infections, iron supplementation, and the use of anti-inflammatory drugs. The participants were instructed to consume a light meal consisting of protein, carbohydrates, and limited fat (such as cereal with milk) on the day of the study. The meal had to be consumed at least 2 h before the beginning of the examination. All athletes belonged to the youth groups of ENEA AZS AJP Gorzów Wielkopolski (1st League and Premier League) and during the study all followed the same training plan. During the two-week rest period, consisting of ten training units of moderate intensity, athletic-force training was conducted on odd-numbered days (Monday, Wednesday, Friday), lasting 1.5 h per training unit, while training on individual technique was conducted on even-numbered days (Tuesday, Thursday), also lasting 1.5 h per training unit. Weekends were allocated for rest, during which no training activities took place. All participants and, in the case of underage athletes, their parents/legal representatives were fully informed about the protocol before the start of the study and all participants, and their legal representatives/parents gave their written consent. The study was conducted by the principles of the Declaration of Helsinki and approved by the Bioethical Committee at the Poznan University of Medical Science, Poland. Anthropometric characteristics of the study participants are presented in Table 1. Research was conducted at the end of the competitive period, specifically two weeks after the last competition games.

**Table 1:** Anthropometric data, maximal heart rate (HRmax), and exercise performance parameters of participants (n = 27).

Variables	Mean ± SD
Age (years)	16.55 ± 0.96
Body mass (kg)	66.40 ± 13.68
Height (cm)	173.45 ± 5.14
Training internship (years)	7.30 ± 1.20
HR_max_ (Bpm)	192.13 ± 12.29
Test duration (min)	10,57 ± 1,60
V_max_ (km h^−1^)	12.85 ± 1.14
VO_2max_ (ml kg^−1^ min^−1^)	51.46 ± 8.41
RER	0.97 ± 0.11

Data ix presented as the mean ± standard deviation. HRmax, maximal heart rate; Vmax, maximal run velocity; VO2max, maximal oxygen consumption; RER (VCO2/VO2), respiratory exchange ratio.

### Design of the study

During the research participants were engaged in acute exercise until reaching exhaustion.

**Figure 1:**
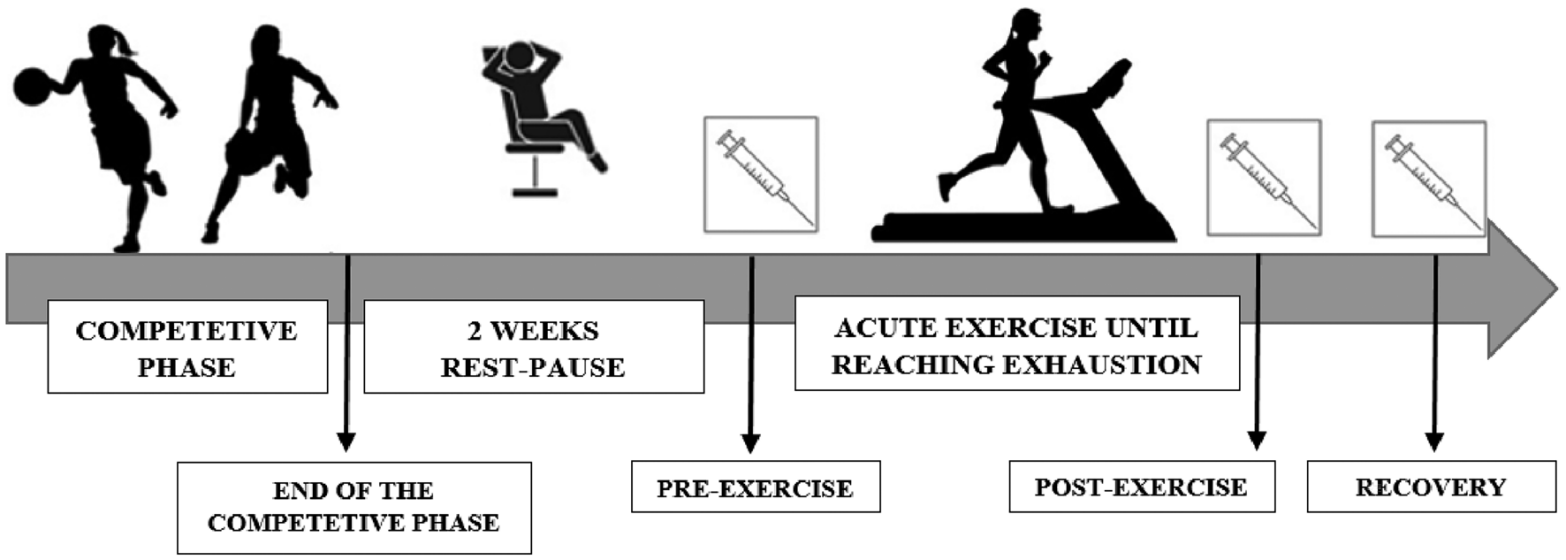
Study design.

Biochemical assays were conducted on blood samples collected at three specific time points: at rest baseline (Pre-exercise), immediately after exercise (post-exercise), and after a 3-h recovery (3 h Recovery) (Fig. 1). An incremental exercise test using the HP Cosmos Treadmill (serial no. cos30004va04; Nussdorf-Traunstein, Germany). Test protocol involved initiating the treadmill at a speed of 8.0 km/h for participants, with subsequent increments of 1.0 km/h every 2 min until reaching exhaustion. Participants fulfilled the exercise test until voluntary exhaustion.

Throughout the exercise test, minute ventilation (VE), oxygen uptake (*V*O_2_), and carbon dioxide production (*V*CO_2_) were continuously measured using the MES exhaled air analyzer (measurement system with a patented pneumotach headpiece by MES and rapid analyzers for carbon dioxide and oxygen, fit for "breath-by-breath" method). Based on the obtained gasometric values the oxygen uptake was calculated for each subject. *V*O_2max_ was also expressed in relative values (ml kg^−1^ min^−1^). Considering the individual variability in physiological responses the highest *V*O_2max_ value obtained during the test and, volitional exhaustion, were used as the criteria used to determine the *V*O_2max_ values considered in the present study. Participants received verbal encouragement to maintain their efforts for as long as possible. Heart rate (bpm) was documented using sports tester (Polar PE 3000) (Table 1).

### Collection of blood and performance of assays

The blood for testing was collected by a qualified phlebotomist using the Sarstedt Safety Monovette system. Polyethylene tubes containing dipotassium ethylenediaminetetraacetic acid (EDTAK2) anticoagulant were used for blood morphology. Polyethylene clotting activator tubes were used for serum obtaining. In this case, the blood was centrifuged to separate the morphotic elements from the serum using a centrifuge (3000 rpm for 10 min). Serum was subsequently frozen at −80 °C until analysis.

Hematological parameters, including white blood cell count (WBC), lymphocyte (LYM), monocyte (MON), and granulocyte (GRA), were examined using the MYTHIC 18 (Orphee Medical, Geneva, Switzerland). Determination of serum levels of total antioxidant capacity (TAC) (Omnignostica Forschungs, Höflein, Austria), superoxide dismutase (SOD), protein carbonyl (PC), thiobarbituric acid reactive substances (TBARS), creatine kinase (CK), IL-1α, IL-2, IL-13, TNFα (SunRed Biotechnology Co., Ltd, Shanghai, China), IL-10 (DRG International Inc., Springfield, New York, USA), and tryptophan (TRP) (LDN, Nordhorn, Germany), were performed using enzyme-linked immunosorbent assay kits and the Thermo Scientific Multiscan GO microplate spectrophotometer (Fisher Scientific, Vantaa, Finland).

### Statistical analysis

All statistical analyses were conducted with STATISTICA v. 13.0 software package (StatSoft Inc., Tulsa, OK, USA). All results are expressed as mean and standard deviation (x ± SD). Normal distribution of variables was assessed using the Shapiro–Wilk test, while Levene’s test was used to examine the homogeneity of variance. Differences in measured variables at the three assessment points (pre-exercise, post-exercise, and 3 h recovery) were evaluated through one-way analysis of variance with repeated measures (ANOVA), followed by Tukey's post-hoc analysis. Cohen's d was calculated as a measure of effect size, with interpretation based on Cohen's criteria: small (0.2), moderate (0.5), and large (0.8). Pearson’s coefficient of linear correlation was computed for correlation analysis. The significance level for all analyses was set at p ≤ 0.05. Based on a power analysis, all tests that yielded significant results had a power above 0.8, as calculated by G Power 3.1.

### Ethics approval and consent to participate

The study was conducted in accordance with the Declaration of Helsinki, and its protocol was approved by the local Ethics Committee at Poznań University of Medical Sciences (decision no. 714/18 of 14 June 2017). All procedures and potential risks were discussed with the participants before the study. Informed consent was obtained from all parents or legal guardians and subjects before participation in the study.

## Results

### Markers of oxidative stress and antioxidant defense

**Figure 2:**
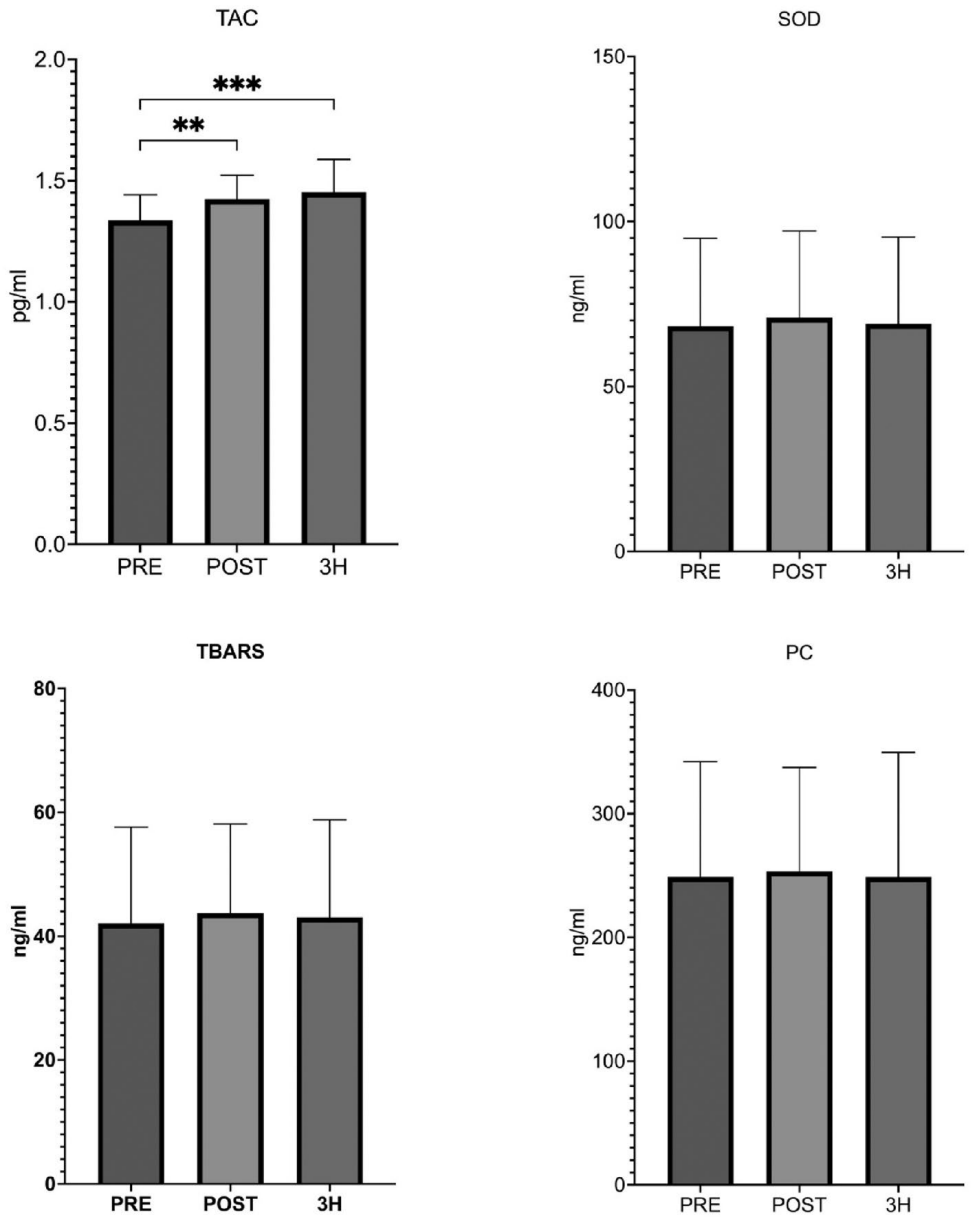
Changes of oxidative stress markers and antioxidant defense during acute exercise. *TAC* total antioxidant capacity, *SOD* superoxide dismutase, *PC* protein carbonyl, *TBARS* thiobarbituric acid reactive substances, *PRE* pre exercise, *POST* post exercise, *3H* 3 h recovery. Significant differences **p < 0.01: ***p < 0.001.

TAC concentration significantly increased after the exercise test (p < 0.05; Cohen's d = 0.90; pre-exercise vs. post-exercise), and even after 3 h of restitution it was still at a significantly high level (p < 0.05; Cohen's d = 1.04; pre-exercise vs 3 h recovery). No significant changes in SOD, PC, and TBARS concentrations were observed. Only trends to their increase were observed immediately after exercise and then decreased after 3 h of restitution (Fig. 2).

### WBC indices

Leukocyte count was significantly increased after exercise (p < 0.05; Cohen's d = 4.76; pre-exercise vs. post-exercise) and significantly decreased after 3 h restitution (p < 0.05; Cohen's d = 2.39; pre-exercise vs 3 h recovery; p < 0.05; Cohen's d = 1.95; post-exercise vs 3 h recovery). Number of lymphocytes significantly increased after exercise and then decreased after 3 h of restitution (p < 0.05; Cohen's d = 4.19; pre-exercise vs. post-exercise; p < 0.05; Cohen's d = 4.11; exercise vs. 3 h recovery) and monocyte count (p < 0.05; Cohen's d = 2.14; pre-exercise vs post-exercise; p < 0.05; Cohen's d = 2.32; post-exercise vs 3 h recovery). Number of granulocytes was significantly increased after exercise, then significantly decreased after 3 h of restitution (p < 0.05; Cohen's d = 2.06; pre-exercise vs. post-exercise; p < 0.05; Cohen's d = 1.89; exercise vs 3 h recovery) (Table 2).

**Table 2:** Changes in WBC indices during acute exercise.

Parameter	Pre-exercise	Post-exercise	3 h recovery	d Cohen		
				Pre-exercise vs post-exercise	Pre-exercise vs 3 h recovery	Post-exercise vs 3 h recovery
WBC (10^3^ µL^−1^)	5.73 ± 0.12	10.65 ± 1.96^a^	7.45 ± 1.32^b,c^	4.73	2.39	1.95
LYM (10^3^ µL^−1^)	1.96 ± 0.41	4.22 ± 0.67^a^	1.88 ± 0.47^c^	4.19	0.18	4.11
MON (10^3^ µL^−1^)	0.39 ± 0.10	0.69 ± 0.18^a^	0.40 ± 0.07^c^	2.14	0.12	2.32
GRAN (10^3^ µL^−1^)	3.37 ± 0.80	5.75 ± 1.51^a^	5.15 ± 1.08^b^	2.06	1.89	0.46

Values are presented as the mean ± SD.

Effect size (Cohen’s d): 0.2 = small; 0.5 = medium; 0.8 = large.

*WBC* white blood cells, *LYM* lymphocytes, *MON* monocytes, *GRAN* granulocytes.

^a^Pre-exercise vs. post-exercise.

^b^Pre-exercise vs. recovery.

^c^Post-exercise vs. recovery (p < 0.05).

### Muscle damage and pro –anti-inflammatory markers

**Figure 3:**
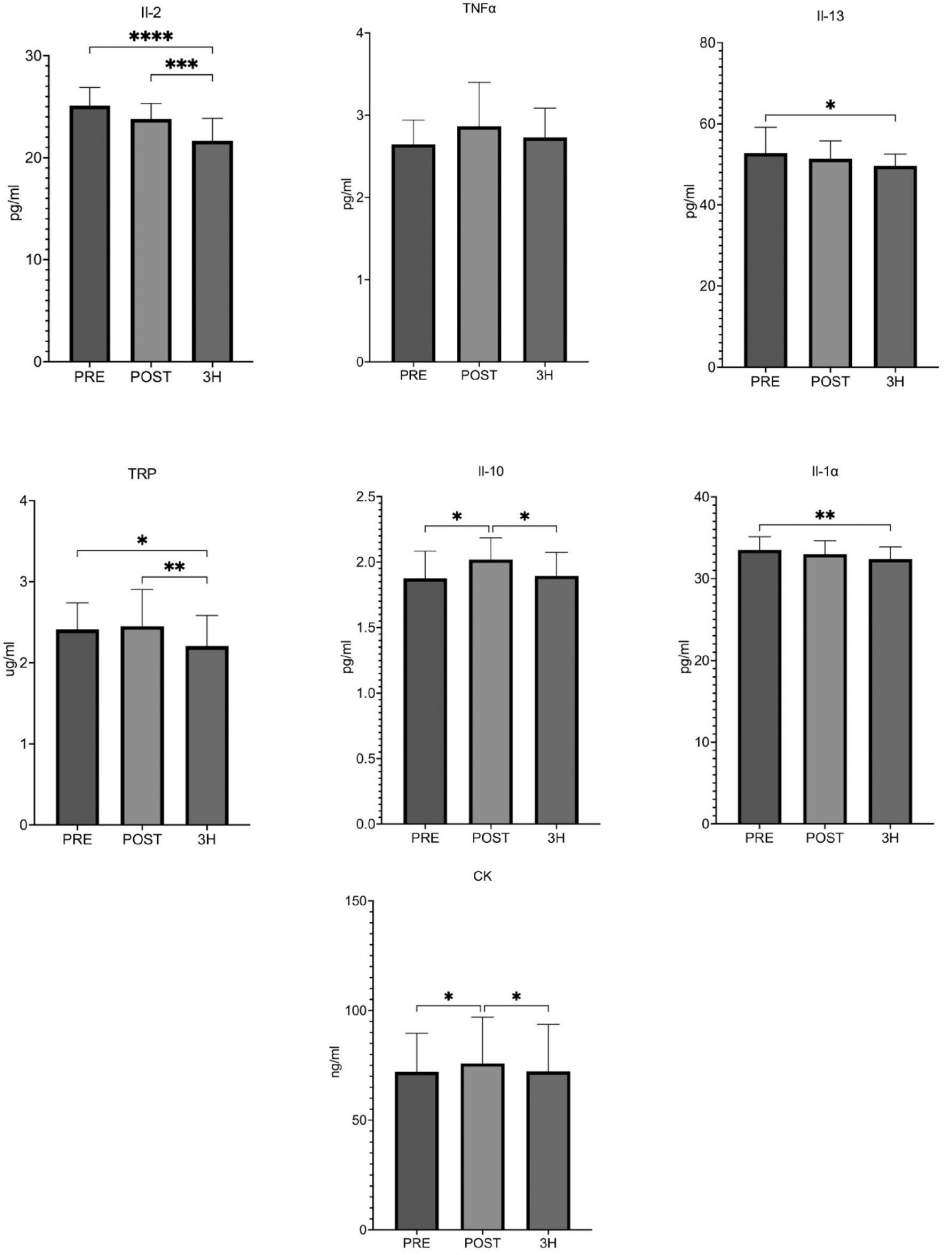
Changes in muscle damage markers and pro-anti-inflammatory balance. *CK* creatine kinase, *Il-1α* interleukin 1α, *TNF α* tumor necrosis factor alpha, *IL-10* interleukin 10, *IL-2* interleukin 2, *IL-13* interleukin 13, *TRP* tryptophan, *PRE* pre-exercise, *POST* post exercise, *3H* 3 h recovery. Significant differences: *p < 0.05 **p < 0.01:***p < .001.

CK was significantly elevated after exercise (p < 0.05; Cohen's d = 0.20; pre-exercise vs. post-exercise) and significantly decreased after 3 h of restitution (p < 0.05; Cohen's d = 0.18; post-exercise vs. 3 h recovery) (Fig. 3).

IL-10 concentration increased significantly after exercise (p < 0.05; Cohen's d = 0.16; pre-exercise vs. post-exercise) and after 3 h of restitution and was significantly decreased (p < 0.05; Cohen's d = 0.15; post-exercise vs 3 h recovery). IL-1α level decreased significantly after 3 h recovery (p < 0.05; Cohen's d = 0.75; pre-exercise vs. 3 h recovery). IL-13 levels significantly decreased after 3 h recovery (p < 0.05; Cohen's d = 0.70; pre-exercise vs. 3 h recovery). IL-2 levels significantly decreased after 3 h restitution (p < 0.05; Cohen's d = 1.77; pre-exercise vs. 3 h recovery; p < 0.05; Cohen's d = 1.19; post-exercise vs. 3 h recovery). No significant changes in the concentration of IL-6 and TNFα were observed. TRP concentration significantly decreased after 3 h of restitution (p < 0.05; Cohen's d = 0.58; pre-exercise vs. 3 h recovery; p < 0.05; Cohen's d = 0.59; post-exercise vs. 3 h recovery) (Fig. 3).

### Correlation

Also, the following positive correlations among markers of oxidative stress were observed: SOD with PC (r = 0.9475; p < 0.0001) and TBARS (r = 0.9308; p < 0.0001). PC with TBARS (r = 0.9119; p < 0.0001) and IL-1α (r = 0.2329; p < 0.05). Moreover, IL-1α was positively correlated with IL-2 (r = 0.3432; p < 0.001). CK was positively correlated with SOD (r = 0.9393; p < 0.0001), PC (r = 0.9058; p < 0.0001), TBARS (r = 0.9358; p < 0.0001), IL-1α (r = 0.2325; p < 0.05) and IL-13 (r = 0.2862; p < 0.001) (Table 3).

**Table 3:** Matrix correlation for study variables.

Variables	CK	SOD	PC	TBARS	IL-1α	IL-10	IL-2
CK							
SOD	0.9393***						
PC	0.9058***	0.9475***					
TBARS	0.9358***	0.9308***	0.9119***				
IL-1α	0.2325*	0.2171	0.2329*	0.2081			
IL-10	0.0547	0.0435	0.0382	0.0700	0.0588		
IL-2	0.0456	−0.0076	−0.0056	0.0092	0.3432**	0.1630	

*CK* creatine kinase, *SOD* superoxide dismutase, *PC* protein carbonyl, *TBARS* thiobarbituric acid reactive substances, *Il-1α* interleukin 1α, *IL-10* interleukin 10, *IL-2* interleukin 2, *IL-13* interleukin 13.

**p < 0.05; **p < 0.001; ***p < 0.0001.

## Discussion

During the season, elite basketball players are exposed to exhausting training loads and tight game schedules, leading to a shorter regeneration time, especially in the starting period. Results published so far have shown that during training periods with the highest loads, muscle contractions increase the stimulation of ROS production in muscle fibers, contributing to the intensification of oxidative stress and in consequence an increase in oxidative damage (e.g., lipid peroxidation and increased protein oxidation). These negative changes may result in disorders in the functioning of the immune system, and increased muscle damage, leading to fatigue or overtraining^9,13^. The presented study aimed to assess whether a two-week period with a low training load is sufficient for full regeneration of female athletes, which may lead to increased adaptation processes.

Reduction of training loads in the investigated basketball players resulted in a significant increase in TAC after intense physical exercise, which was accompanied by no significant changes in the concentration of SOD (Fig. 2). Changes in the TAC concentration observed in our study may confirm the high level of adaptation of female athletes to oxidative stress tolerance, manifested as the strengthening of antioxidant mechanisms that effectively prevented not only the formation of oxidative stress but also the harmful effects of free radicals^14,15^. Available literature data suggests that intense physical exercise contributes to the increase in ROS leading to oxidative stress^16^. However, we obtained similar results to Bessa et al. (2016)^17^, not observing significant changes in the levels of antioxidant enzymes and markers of oxidative damage (e.g., TBARS). They studied the changes in markers of oxidative stress in cyclists performing a combination of high-intensity aerobic and anaerobic exercise, preceded by a week-long break from physical activity. Observed changes were explained by the fact that antioxidant enzymes are regulated by the redox status, and not by physical exercise alone^18^. High resting levels of antioxidant enzymes observed in athletes probably contribute to the fact that ROS production in response to exercise is not sufficient to change the redox status. Moreover, Spanidis et al. (2015)^13^ studied professional basketball players at the beginning (9 games) and at the end (59 games) of the league season, checking the impact of match loads and those related to the number of matches on parameters related to oxidative stress. Authors showed that the highest number of matches at the end of the season caused stronger oxidative stress compared to the first phase. Therefore, it can be concluded that the accumulation of training loads together with the lack of rest negatively affects the parameters related to oxidative stress.

Intense physical effort performed by rested female athletes did not significantly affect the change in the concentration of TBARS and PC (Fig. 2). Obtained results may confirm the effective activation of adaptive responses to oxidative stress^13^. They also show maintained redox balance after intense physical exercise in rested athletes, which cannot be observed in those in the starting period when training loads are the highest^18,19^. Therefore, it seems that an important factor influencing the level of damage is the phase of the training cycle when the exercise tests were performed, as well as the type of exercise. Results of elite basketball players' studies indicate that at the end of the season, oxidative stress induced by physical exercise is not strong enough to lead to lipid peroxidation^13^. This is explained by the increasing efficiency of mechanisms of antioxidant defense through the thiol status-regulated pathway (i.e., reducing the level and oxidation of GSH)^13^. Also, the available literature shows a sharp increase in PC concentration in athletes in response to intense physical effort^20,21^. Studies in which no changes or a decrease in PC concentration were observed show that the redox balance may be maintained after physical exercise, even when other markers of oxidative stress (e.g., markers of lipid peroxidation) are increased^18,22^.

Physical effort is also a factor causing microdamage in the sarcolemma, increasing muscle damage indicators in blood, such as CK. The increase in CK may also be due to immune and hormonal changes induced by high-intensity training. In addition, it has been proven that the CK response to exercise depends on the time of intensity, length, and even the type of sports discipline^19,23,24^.

In our study, an increase in CK was observed immediately after the exercise test, the value of which returned to baseline after 3 h of restitution (Fig. 3). At the same time, a correlation of CK with both pro-inflammatory cytokines and parameters related to oxidative stress was observed, which may indicate that in the period when the athletes are rested, the production of pro-inflammatory cytokines is related to the increase in muscle fiber damage and the related stimulation of ROS production. Observations of CK concentrations in relation to the training phases of the annual cycle allowed us to observe a higher increase in CK induced by physical exercise before the competition, compared to the preparation period, which was probably due to higher intensity and more specialized exercises, leading to increased muscle damage caused by physical exercise or body adaptation for exercise^25^. Our results stand in contrast to the ones obtained by Bachero-Mena et al. (2017)^26^, who found no significant changes in CK during the whole season in middle-distance runners.

Available literature indicates a significant role of ROS and muscle fiber damage in the release of cytokines into the bloodstream in response to exercise, but the mechanism is still unclear^27^. Exercise-induced inflammatory response is manifested by marked leukocytosis, which is mainly due to the release of lymphocytes, monocytes, and neutrophils into the circulation.^28,29^.

In tested basketball players, there was a clear leukocytosis under the influence of physical effort, which confirms the presence of post-exercise inflammation. In addition, we showed transient lymphocytosis, monocytosis, and granulocytosis immediately after exercise (Table 2). All the described changes in the number of leukocyte cells are probably caused by their increased recruitment from the bone marrow because of muscle fiber damage^30^. It should be assumed that the adaptation mechanisms and regeneration contributed to a reduction in the number of leukocyte cells within 3 h after exercise. Literature data indicate that transient lymphocytosis and monocytosis occur first immediately after exercise, followed by delayed neutrophilia after 2 h of recovery. In addition, Da Silva Neves et al.^31^ showed that acute and short-term physical exercise causes a greater increase in the number of leukocytes than low-intensity exercise.

It is expected that the release of cytokines is associated with many factors, such as the intensity, duration of exercise, and the training of athletes^32^. It was also indicated that long-term endurance exercise causes a strong response of pro-inflammatory cytokines, while in the case of short-term intense exercise, changes in their concentration are negligible. However, there are few studies explaining whether the rest from exercise exerts an immunomodulatory effect on the production of inflammatory cytokines during periods of lighter training loads.

In our studies, we did not observe significant changes in TNF-α levels after a session of intense exercise (Fig. 3). The reason may lie in the adaptation of athletes to physical effort as well as the reduction of high-intensity exercise during this period^33,34^. Lack of an increase in the concentration of pro-inflammatory cytokines with a simultaneous high increase in WBC may also confirm the fact that lymphocytes and monocytes are not responsible for the rapid increase in pro-inflammatory cytokines in response to exercise. Moldoveanu et al. (2000)^35^ reached a similar conclusion, as they did not observe changes in gene expression in circulating mononuclear cells, which may indicate other sources of pro-inflammatory cytokines. Bay and Pedersen (2020)^36^ indicate that in such cases the inflammatory response is also regulated by the production of ROS and contracting muscle fibers. Therefore, it seems that slight disturbances in the pro/antioxidant balance demonstrated after a single refusal test in rested athletes contributed to the lack of a rapid release of pro-inflammatory cytokines.^37^.

Remaining pro-inflammatory cytokines in our studies either did not change their concentration, as in the case of IL-1 α, or even their level decreased after exercise, as in the case of IL-2 (Fig. 3). In their research, Jürimäe et al. (2018)^38^ did not observe significant changes in IL-1α levels after one hour of endurance training. On the other hand, Mezil et al. (2015)^39^ observed an increase in IL-1α immediately after exercise and then showed a significant decrease within an hour. These changes were accompanied by an immediate increase in IL-6 and TNF-α followed by a decrease, which we did not observe in our study, which may indicate the reason for the unchanged production of the cytokine IL-1α. Research results also indicate reduced IL-2 production in response to exercise^35,40^. Baum et al. (1997)^41^ observed no changes in IL-2 levels. This varied state of knowledge seems to result from different types of training, as well as the state of training of athletes. Observed significant decrease in IL-2 concentration in our study may be the result of either the suppression of cellular immunity because of exercise, or the effect of anti-inflammatory cytokines.

Level of anti-inflammatory IL-10 significantly increased after the exercise test in our female athletes, with a simultaneous decrease in IL-13 during the restitution period (Fig. 3). The response of IL-10 to physical effort depends on the athletes' level of training and practicing discipline. A significant increase in IL-10 concentration was observed in endurance-trained people compared to sprint-trained.^42^. In addition, in the period of immediate preparation for the competition, when the athletes were subjected to the greatest training loads, an increase in IL-10 was shown with no changes in its concentration during the rest period from training. Research results indicate that physical exercise causes a significant increase in IL-13 in non-training people (Tufvesson et al. (2020)^43^ and Alizadeh et al. (2019)^44^), on the other hand, Zamani et al. (2014)^45^ showed no significant differences in the concentration of IL-13 in elite wrestlers 24 h after training and in the control group. Observed effect of intense physical exercise on anti-inflammatory cytokines in our study may indicate a high effort tolerability and undisturbed activity of the immune system reacting with rapid activation of anti-inflammatory processes (anti-inflammatory effect of exercise).

It is presumed that exercise affects the concentration of TRP, a precursor of serotonin and kynurenine, which may be related to fatigue in response to training^46–48^. In our study, the level of TRP significantly decreased and its reduction was observed even after 3 h of restitution (Fig. 3). State of knowledge about changes in TRP concentration is inconsistent. Kreher et al. (2012)^1^ showed a positive correlation between TRP increase and fatigue. On the other hand, Joisten et al. (2020)^49^ showed in their studies that endurance exercise causes a significant decrease in TRP concentration immediately after physical exercise, and, after 1 h, the concentration would return to its baseline values. Immediately after resistance exercise, however, a trend of a decrease in TRP concentration has been observed, and its further reduction even after an hour of rest. Still, Ikonen et al. (2020)^50^ did not observe changes in its concentration and pointed out that it is not a sensitive marker of changes associated with overload. This may indicate the need to extend the overload diagnostics in subsequent tests with additional parameters. In addition, there is a hypothesis stating that reduced TRP levels accompany systemic inflammation, which is explained by using the amino acid for the synthesis of inflammation-related proteins. It is also used by leukocytes, which may explain the decrease in TRP concentration in our study^1,51^.

It is worth noting that the study had some limitations, like an important aspect that the results were obtained only when the athletes were rested. No comparison was made with the period when training loads were the highest. Future research should include performing the test at a time when training loads are high, as well as when the athletes are rested. Additionally, a limitation of the study is the lack of consideration of the phase of the menstrual cycle, which could have influenced the tested parameters. Despite the above-mentioned limitations, the study enriched our knowledge about the impact of two-week regeneration on the parameters of oxidative stress and inflammation.

## Conclusion

Obtained results show that the applied exercise protocol did not generate oxidative stress in athletes, despite previous reports that intense exercise is a strong stimulator of ROS production. At the same time, the strong effect of the antioxidant potential is visible, eliminating the negative effects of post-exercise oxidative stress and the post-exercise inflammatory response. The study results may indicate that limiting high-intensity exercise for 2 weeks contributed to the athletes' better tolerance to the applied physical effort. Obtained information regarding the rate of adaptation and exercise tolerance preceded by a period of rest will require further detailed research. Their results can help plan the optimization of training processes, especially in the period of the greatest effort loads, to minimize the phenomenon of fatigue and overtraining.

The study results may indicate that limiting high-intensity exercise for 2 weeks contributed to the athletes' better tolerance to the given physical effort. Gathered information regarding the rate of adaptation and exercise tolerance preceded by a period of rest will require further detailed research. Their results can help plan to optimize training processes, especially in the period of the greatest effort loads, to minimize the phenomenon of fatigue and overtraining.

This study recommends that coaches consider withdrawing an athlete from competition if they observe symptoms of fatigue to minimize the risk of overtraining. Rested athlete can then undergo a full regeneration process, which may lead to positive effects in the form of improved sports results. This action is consistent with the principles of optimizing sports performance and caring for the health and physical condition of players.

## Data Availability

Due to ethical concerns the datasets generated and/or analyzed during the current study supporting data cannot be made openly available however, are available from the corresponding author on reasonable request.
